# Trunk dental tissue evolved independently from underlying dermal bony plates but is associated with surface bones in living odontode-bearing catfish

**DOI:** 10.1098/rspb.2017.1831

**Published:** 2017-10-18

**Authors:** Carlos J. Rivera-Rivera, Juan I. Montoya-Burgos

**Affiliations:** 1 Department of Genetics and Evolution, University of Geneva, Geneva, Switzerland; 2 Institute of Genetics and Genomics in Geneva (iGE3), University of Geneva, Geneva, Switzerland

**Keywords:** trunk neural crest, Loricarioidei, odontogenesis, Siluriformes, phylogenetics, dental tissue

## Abstract

Although oral dental tissue is a vertebrate attribute, trunk dental tissue evolved in several extinct vertebrate lineages but is rare among living species. The question of which processes trigger dental-tissue formation in the trunk remains open, and would shed light on odontogenesis evolution. Extra-oral dental structures (odontodes) in the trunk are associated with underlying dermal bony plates, leading us to ask whether the formation of trunk bony plates is necessary for trunk odontodes to emerge. To address this question, we focus on Loricarioidei: an extant, highly diverse group of catfish whose species all have odontodes. We examined the location and cover of odontodes and trunk dermal bony plates for all six loricarioid families and 17 non-loricarioid catfish families for comparison. We inferred the phylogeny of Loricarioidei using a new 10-gene dataset, eight time-calibration points, and noise-reduction techniques. Based on this phylogeny, we reconstructed the ancestral states of odontode and bony plate cover, and find that trunk odontodes emerged before dermal bony plates in Loricarioidei. Yet we discovered that when bony plates are absent, other surface bones are always associated with odontodes, suggesting a link between osteogenic and odontogenic developmental pathways, and indicating a remarkable trunk odontogenic potential in Loricarioidei.

## 1. Introduction

A skeletal system formed by mineralized tissues is a major vertebrate innovation that contributed to the evolutionary success of this lineage [1]. Dermal bones and dental structures are important components of this skeletal system and can be found, often in direct contact, in almost all extinct and extant gnathostomes (jawed vertebrates) [1–5]. However, while dermal bones have formed part of the skeleton of both the head and the trunk throughout vertebrate evolution, dental structures, although present in the body in some extinct lineages, are found almost exclusively in the head in extant species.

The rarity of trunk dental tissue in extant vertebrates has been explained by the reduced potential of neural crest (NC) cells in the trunk relative to their potential in the head [6]. The NC is an ectodermal cell lineage exclusive to vertebrates that is required for the production of a variety of cell types [7], dental structures and some cranial bones [5,8]. Currently, there are two recognized subpopulations of NC cells with different potentials and fates: cranial and trunk NC cells [9]. Several studies have found that the trunk NC has a reduced developmental potential relative to the cranial NC *in vivo* [10–12], and one of the potentials presumed to be lost is that of producing bony and dental structures [13,14].

However, there are exceptional living vertebrate lineages that have dental structures on their trunk. These extra-oral dental structures are called odontodes, and they are virtually indistinguishable from oral teeth at the structural level (they contain dentine, enamel and a pulp cavity). Odontodes were also an integral part of the dermal bony armour of many lineages of extinct jawed and jawless vertebrates [15]. Interestingly, in both extinct and extant vertebrates, trunk odontodes are always associated with an underlying dermal bony plate. This general rule seems to apply to all known lineages, and the few instances in which trunk odontodes are found without an underlying dermal bony plate are cases in which these bony plates were secondarily reduced, as in chondrichthyans (sharks and rays) [16].

These observations led us to investigate whether unlocking odontogenesis in the trunk requires the pre-existence or the concomitant emergence of dermal bony plates in the trunk, even if the plates are later reduced or lost while trunk odontodes are retained. A consistent pattern of evolutionary association between trunk dermal bony plates and dental elements in fossil and extant lineages would suggest an interplay between the activation of osteogenic gene pathways and the potentialization of trunk tissues for odontogenic fates. If this were the case, we would expect that all species with trunk odontodes would have ancestors with trunk dermal bony plates with or without odontodes.

**Figure 1.**
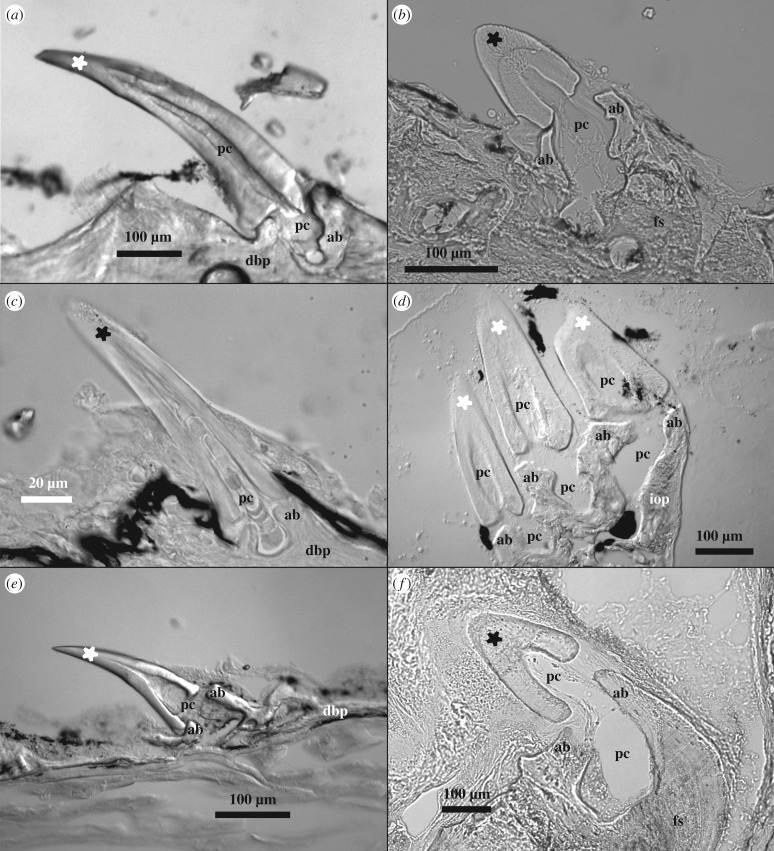
Light microscopy images using Nomarski interference contrast of 8 µm thick histological cryosections of trunk odontodes in representatives from all six families of Loricarioidei. (*a*) An odontode on a dermal bony plate of a representative of the Loricariidae family, *Planiloricaria cryptodon*. (*b*) An odontode on the caudal fin spine of a member of Astroblepidae, *Astroblepus* sp. (*c*) An early odontode on a dermal bony plate of a juvenile from the Callichthyidae family, *Corydoras sterbai*. (*d*) Three odontodes growing on the interopercle bone of *Tridensimilis brevis*, a member of Trichomycteridae. (*e*) An odontode growing on a dermal bony plate from the ventral series of a member of the Scoloplacidae family, *Scoloplax* sp. (*f*) An odontode growing on the pectoral fin spine of the only member of the Nematogenyidae family, *Nematogenys inermis*. The crown of each individual odontode is marked by a star, and the abbreviated terms are the following: pulp cavity (pc), attachment bone (ab), dermal bony plate (dbp), interopercle bone (iop), and fin spine (fs). Black spots in the images are from skin pigmentation.

The catfish order (Teleostei: Siluriformes) provides a living case study for addressing this question, as members of this lineage possess both trunk dermal bony plates and trunk odontodes in different combinations (figure 1). This order is organized into three groups: the South American Diplomystidae family, the globally distributed Siluroidei suborder and the South American Loricarioidei suborder [17–20]. All species of Siluriformes lack scales, and the assumed ancestral state of the order is naked skin [21]. However, some groups within this order independently evolved dermal bony plates that cover their bodies to different degrees. While in most lineages these trunk dermal bony plates are only composed of bone [22], within the Loricarioidei suborder, these plates bear odontodes [23] (figure 1). These odontodes seem to have different functions across species, and they may be important in territorial disputes, provide better hydrodynamics in rheophilic species and serve as protection against predation in juveniles (personal observations). Loricarioid odontodes have been studied in detail by other authors [22,24–29], but these studies were focused on location and structure in selected representatives.

The cover and location of both bony plates and odontodes vary greatly from family to family in the Loricarioidei, with some displaying complete cover of the body surface with plates bearing odontodes and others lacking bony plates altogether with odontodes on some fins only. Tracing the evolution of these characters in the Loricarioidei phylogeny would allow us to determine the pattern of trunk odontode emergence, and through ancestral state reconstruction we can infer whether the formation of these trunk odontodes was preceded by the emergence of trunk dermal bony plates in the ancestor to all Loricarioidei. Answering this question using an extant lineage in which trunk odontodes emerged relatively recently, rather than using an extinct fossil lineage, provides us with a more comprehensive view of the evolutionary paths that led to the origination of these characteristics.

The reconstruction of ancestral traits requires a robust, time-calibrated phylogeny and a precise knowledge of the character states in the terminal extant taxa. The phylogenetic relationships among loricarioid families have been addressed with both morphological [17,19,24,30–32] and molecular data [18,33], but there is conflict among the inferred hypotheses of relationships. The published molecular studies used relatively small datasets (one and two genes), and show signs of a lack of information and phylogenetic artefacts that emerge due to substitution saturation [18,33]. Because a robust phylogeny is required to enable a realistic reconstruction of the ancestral condition of the Loricarioidei, a more thorough analysis with a larger molecular dataset must be conducted.

For this study, we first collected information on the presence and location of trunk dermal bony plates and odontodes for all loricarioid families and several outgroup catfish families. Then, using representatives from the same groups, we produced a new 11 188 bp multi-gene sequence dataset consisting of 10 genes sequenced in 47 taxa. We performed a thorough phylogenetic analysis of this dataset and confirmed old and proposed new inter-familial arrangements within the Loricarioidei. We dated the age of the families by time calibrating the phylogeny using eight calibration points. Based on the character state information and the time-calibrated phylogeny, we reconstructed the ancestral condition of trunk dermal bony plates and odontodes in all inter-familial loricarioid ancestors, and showed how these trunk skeletal and dental structures were associated during Loricarioidei evolution.

## 2. Material and methods

### (a) Taxon sampling and data collection

We studied all six families of Loricarioidei, with 17 other non-loricarioid catfish families as outgroups. Within the outgroups, we included all four siluriform families that are known to contain species with dermal bony plates (without odontodes) on their trunk.

Our morphological scoring was based on the information contained in the extensive literature on siluriform morphology, and we confirmed the states with living or museum specimens, when these were available. For trunk dermal bony plates, we used a binary character to reflect the presence or absence of dermal bony plates on the species in the family. In the literature, a specimen is determined to have a bony plate when osseous plates of dermal bone are found on the skin surface (e.g. [24]). For odontodes, we plotted a four-state character to represent odontode location: no odontodes (0), odontodes on the head but not on the trunk (1), odontodes somewhere on the trunk but not on the head (2), and odontodes on the head and on the trunk (3). In the literature, odontodes are usually defined as extra-oral structural elements that have a crown, a pulp cavity and an attachment bone, and in which dentine can be observed as an electron-dense mineralized tissue when observed with transmission electron microscopy (e.g. [22,26]). Our unit of analysis was the family; therefore, we coded the presence or absence of a character at the family level (i.e. when the character was present in at least one representative, it was coded as present in the family). The museum specimens were provided by the Museum of Natural History of Geneva (MNHG) (voucher numbers and reference numbers in electronic supplementary material, table S1). For illustrative purposes, we also extracted odontode-bearing tissues from representative species of each of the six loricarioid families and did histological cryosections and imaging using Nomarski interference contrast (methods in electronic supplementary material).

The molecular dataset included 47 representatives of the 23 families from the morphological study, 21 from Loricarioidei and 26 outgroups. DNA samples came from the DNA collection of the MNHG (voucher numbers in the electronic supplementary material, table S2). We used a total of 10 genes, four of which were new genetic markers for inferring inter-familial relationships in Siluriformes: the 28S large ribosomal subunit (LSU), *rhodopsin*, fish reticulon 4 receptor-like 2a (*rtn4rl2a*) and periplakin (*ppk*). We also sequenced six markers that are frequently used in fish phylogenetics (*rag1, rag2, cytB,* COI, 12S and 16S), and, when available, we took sequences from GenBank. The GenBank accession numbers for these sequences are in the electronic supplementary material, table S3. Genes were amplified and sequenced following standard protocols (electronic supplementary material, information and table S4). Missing sequence data were encoded as question marks.

### (b) Phylogenetic analyses

After checking the sequencing quality on the chromatograms, we aligned by eye the sequences of all markers using BioEdit v. 7.0.5.3 [34] and removed areas of ambiguous alignment. We used MEGACC, the command line implementation of MEGA 7 [35,36], to calculate the best nucleotide substitution model for each gene; for the six coding genes, we also calculated the best amino acid substitution model.

We concatenated the nucleotide sequences of all 10 genes, producing the ‘DS1’ dataset. To determine whether substitution saturation was affecting our inferences, we produced three more versions of this alignment in which we employed increasingly stringent substitution saturation-reduction techniques. In the first instance, we translated all six protein-coding genes into amino acids and kept the four ribosomal RNA genes as nucleotide data, which resulted in a mixed data matrix referred to as ‘DS2’. Translating codons reduces the effect of substitution saturation due to multiple synonymous mutations [37]. For the other two alignment versions, we used the translated protein-coding genes; we then analysed the ribosomal RNA genes' nucleotide data with the *baseml* program of the PAML v. 4.7 package [38] under 10 gamma rate categories (GRC) to assign each site to one of the 10 GRC. We then removed the nucleotide sites of the RNA genes that belonged to the fastest-evolving GRC (category 10; 5.4% of sites) to produce the next alignment, referred to as ‘DS3’, and the nucleotide sites that belonged to the two fastest-evolving GRCs (categories 9 and 10; 10% of sites) to produce the last alignment version, referred to as ‘DS4’. While removing sites results in shorter alignments, these datasets contain less phylogenetic noise due to substitution saturation and a lower potential of containing misleading information [39].

We performed maximum-likelihood (ML) phylogenetic inferences with RAxML v. 8.0.26 [40] and Bayesian inferences (BI) with MrBayes v. 3.2.6 [41], partitioning the data by gene. To test node support in the ML inferences, we performed 1000 bootstrap replicates for each analysis. The BI analyses were performed with 4 chains, 20 million generations sampled every 100th generation and a burn-in of the 25% initial generations.

### (c) Inferring the time-calibrated phylogeny

We produced a time-calibrated phylogeny based on the DS4 alignment (a mixed amino acid and nucleotide matrix with the fast-evolving nucleotide sites belonging to GRC 9 and 10 removed) using eight calibration points. Seven of these points were previously published, and one of them is new in this study (electronic supplementary material, table S5). The new calibration point determines the age of the most recent common ancestor (MRCA) of the clade that includes the loricarioid families Scoloplacidae, Astroblepidae and Loricariidae at a maximum of 100 Mya. This maximum age is based on the observation that all known extant and fossil members of Loricarioidei are South American, and given the large diversity and adaptability of this group, their main radiation must have occurred after the separation of South America and Africa (approx. 100 Ma [42,43]); otherwise, Loricarioidei representatives would be present in Africa, which is not the case according to current knowledge on extant and fossil fishes. However, the internal branches within the most basal groups of Loricarioidei are long and our taxon sampling is not dense enough to determine when the diversification of the species-rich Trichomycteridae and Callichthyidae families occurred. Hence, a conservative way to include this information was to place this calibration point after the emergence of the Trichomycteridae and Callichthyidae and just before the diversification of the Astroblepidae, Scoloplacidae and Loricariidae.

We set the oldest boundary of all the calibration points that lacked a clear oldest possible age at 161.2 Ma, following the maximum age constraint for the clade Otocephala (of which Siluriformes forms part) employed by [44]. We then used BEAUti and BEAST v. 1.8.3 [45] to produce the time-calibrated phylogeny (details in electronic supplementary material).

### (d) Ancestral state reconstruction

We independently reconstructed the ancestral loricarioid conditions for the presence of trunk dermal bony plates and of odontodes on different parts of the body. We analysed each character independently through ML using BayesTraits v. 3.0 [46] based on our time-calibrated tree. All ML runs were repeated 10 000 times (parameter ‘MLT 10000’), and the ancestral state for each family and inter-familial relationship was computed. An in-house bash script parsed the BayesTraits results, and another in-house script in R [47], using the *ape* library [48], plotted the probability of each state as a pie chart on each node of the phylogeny.

We tested two models for the reconstruction of the ancestral condition of odontodes in Loricarioidei. The first model, model 1, assumes that the probability that the ancestral loricarioid gained odontodes is the same if these structures emerged on the head only, on the trunk only or on the full body (*p*_01_ = *p*_02_ = *p*_03_). In addition, this model assumes that the probability of losing the odontodes, either located on the head only, on the trunk only or on the full body, is equal as well (*p*_10_ = *p*_20_ = *p*_30_). Furthermore, all transitions between incomplete odontode cover and a more complete cover are equally probable (*p*_23_ = *p*_31_ = *p*_32_ = *p*_13_). The second model, model 2, focuses more on the emergence of trunk odontogenesis, and hence the probabilities of gaining odontodes on the trunk are the same, regardless of the initial state (*p*_02_ = *p*_03_ = *p*_12_ = *p*_13_). This model also assumes that the probabilities of losing trunk odontodes are the same, regardless of the final state (*p*_20_ = *p*_30_ = *p*_21_ = *p*_31_).

The model used for the inference of the ancestral state of dermal bony plates assumes that the probabilities of gaining and losing plates are independent.

## 3. Results

### (a) Morphological characters

**Figure 2.**
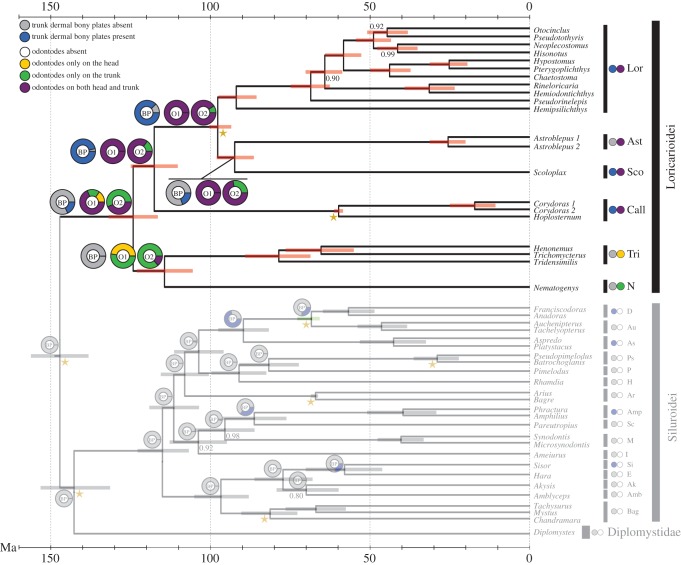
Time-calibrated phylogeny of the Loricarioidei. Node splits are placed at the mean node height, and node bars represent the node height highest posterior density interval at 95%. Stars identify the time calibration points used to obtain this tree, and posterior probabilities lower than one are shown. The observed state of dermal bony armour and odontode cover is shown for each family as two coloured circles beside the bar that identifies the group. Grey on the first circle indicates lack of trunk dermal bony plates and blue indicates presence. On the second circle, white indicates lack of odontodes in the family, yellow indicates odontodes present only in the head, green indicates odontodes present only in the trunk, and violet indicates odontodes present on both head and trunk. Note that there are no families with odontodes in the outgroup. The same colour code is used to represent the probable states of the ancestors. The doughnut chart labelled with ‘BP’ shows the probabilities for the ancestral state of bony plates. The doughnut chart labelled ‘O1’ shows the probable ancestral states of odontode cover obtained under model 1, in which the probabilities of gaining odontodes are the same, regardless of the region in which they emerge, and the doughnut chart labelled with ‘O2’ shows the results of the probable ancestral states of odontode cover as obtained under model 2, in which the probabilities of gaining odontodes in the trunk are different from those of gaining odontodes only in the head. Lor, Loricariidae; Ast, Astroblepidae; Sco, Scoloplacidae; Call, Callichthyidae; Tri, Trichomycteridae; N, Nematogenyidae; D, Doradidae; Au, Auchenipteridae; As, Aspredinidae; Ps, Pseudopimelodidae; P, Pimelodidae; H, Heptapteridae; Ar, Ariidae; Amp, Amphiliidae; Sc, Schilbeidae; M, Mochokidae; I, Ictaluridae; Si, Sisoridae; E, Erethistidae; Ak, Akysidae; Amb, Amblycipitidae; Bag, Bagridae.

Based on the literature and our observations of living and museum specimens, we assembled a character matrix for the presence or absence of dermal bony plates on the trunk in Siluriformes (figure 2; electronic supplementary material, table S6). Trunk dermal bony plates can be found in several lineages across the Siluriformes and, among the groups we considered in this analysis, are present in the Amphiliidae, Aspredinidae, Callichthyidae, Doradidae, Loricariidae, Scoloplacidae and Sisoridae.

We also assembled a character matrix for the presence or absence of odontodes and their location on the body (figure 2; electronic supplementary material, table S6). Only our group of interest, the Loricarioidei, have representatives possessing odontodes (figure 1), but only a subset of loricarioid families have odontodes on the trunk: the Astroblepidae (figure 1*b*), Callichthyidae (figure 1*c*), Loricariidae (figure 1*a*), Nematogenyidae (figure 1*f*) and Scoloplacidae (figure 1*e*). There is one loricarioid family without trunk odontodes, the Trichomycteridae, in which the odontodes are restricted to the opercle and interopercle bones of the head (figure 1*d*).

When both character matrices are compared, different combinations of both traits can be observed on the trunk: only dermal bony plates in Amphiliidae, Aspredinidae, Doradidae and Sisoridae; trunk dermal bony plates and trunk odontodes in Callichthyidae, Loricariidae and Scoloplacidae; and trunk odontodes but no trunk dermal bony plates in Astroblepidae and Nematogenyidae.

### (b) Loricarioidei phylogeny

To infer the phylogeny of the Loricarioidei, we generated a 10-gene sequence alignment of 11 188 nucleotide sites (DS1), which contained a total of 21% missing data. The best substitution models according to the Bayesian information criterion (BIC) are presented in the electronic supplementary material, table S7 for all genes both in their nucleotide and in their amino acid encoding.

The ML analysis of the first nucleotide dataset, DS1, resulted in a topological arrangement in which the oldest split separates a clade composed of the Trichomycteridae and Callichthyidae families, and on the other side, a clade composed of Nematogenyidae followed by the Scoloplacidae and Astroblepidae families sister to Loricariidae (electronic supplementary material, figure S1). However, the bootstrap supports (BS) of almost all inter-familial relationships were low (table 1). The ML and BI inferences differ in that the latter showed the Nematogenyidae as the first group to branch out in Loricarioidei, followed by Trichomycteridae, then Callichthyidae, and finally the Scoloplacidae plus Astroblepidae families sister to Loricariidae, albeit also with low posterior probabilities (PP) (electronic supplementary material, figure S1). The grouping of the Callichthyidae and Trichomycteridae families in the ML inference appears to be the result of phylogenetic artefacts, as both groups have very high substitution rates; based on previous knowledge of these families, this relationship is highly unlikely (see [49]). Indeed, with the second dataset, DS2, where the coding nucleotide sequences were translated into amino acids to reduce noise due to synonymous substitution saturation, this relationship was not present, and both ML and BI inferences agreed in the tree topology. A major change in this new topology was the grouping of the Trichomycteridae with the Nematogenyidae, with good statistical support (BS = 69; PP = 1). In addition, the clade composed of Callichthyidae, Scoloplacidae, Astroblepidae and Loricariidae (the CSAL clade) went from not recovered in the ML analyses of DS1 to recovered with BS = 68 with DS2, and in the BI analyses from PP = 0.83 with DS1 to a PP = 1 with DS2 (table 1). Support for the clade grouping Astroblepidae and Scoloplacidae also increased from a BS of 66 with DS1 to 86 with DS2 and a PP of 0.96 to 1, respectively. For the DS3 dataset, in which the GRCs of the fastest-evolving nucleotide sites were removed to reduce a step further the phylogenetic noise due to substitution saturation, the topology obtained was the same as with DS2, and the statistical support for most of the inter-familial relationships improved or remained high (table 1). The sole exception was the BS for the clade grouping Astroblepidae and Scoloplacidae, which exhibited a slight drop from 86 to 72, but the PP barely changed, from 1 to 0.99. The topology did not change in the analysis of the DS4 dataset, in which the next category of fast-evolving nucleotide sites of the ribosomal genes was additionally removed to further reduce phylogenetic noise. Again, the support for all inter-familial relationships either remained high or increased (table 1).

**Table 1.** Summary of node supports for the monophyly of Loricarioidei and of interfamilial relationships through a series of datasets in which the causes of potential phylogenetic signal saturation are progressively reduced. BS, bootstrap supports after 1000 bootstrap replicates; PP, Bayesian posterior probability after 20M generations; n/r, clade not recovered. N, Nematogenyidae; T, Trichomycteridae; C, Callichthyidae; S, Scoloplacidae; A, Astroblepidae; L, Loricariidae. The last row shows the PP of each clade based on our final, time-calibrated phylogeny as obtained with a 30M generation analysis with BEAST, based on the DS4 dataset and eight time-calibration points.

	Loricarioidei		N + T		CSAL		SAL		S + A	
	BS	PP	BS	PP	BS	PP	BS	PP	BS	PP
DS1	100	1	n/r	n/r	n/r	0.8	100	1	66	1
DS2	100	1	69	1	68	1	99	1	86	1
DS3	100	1	74	1	73	1	100	1	72	1
DS4	100	1	77	1	74	1	99	1	71	1
DS4 + time cal.	—	1	—	1	—	1	—	1	—	1

The best overall statistical node supports for inter-familial loricarioid relationships were obtained with the DS4 dataset. In this phylogeny (electronic supplementary material, figure S1), the Loricarioidei were monophyletic with BS = 100 and PP = 1. The clade was organized as follows: Nematogenyidae and Trichomycteridae clustered together (BS = 77; PP = 1) and were sister to the CSAL clade (BS = 74; PP = 1). Within the CSAL, the Callichthyidae was the first group to branch out, followed by a clade composed of the Scoloplacidae, Astroblepidae and Loricariidae families (BS = 99; PP = 1). This group then divided into the Loricariidae family sister to a clade including the Astroblepidae and Scoloplacidae (BS = 71; PP = 0.99). In addition, this phylogenetic arrangement of Loricarioidei was confirmed, and the supports increased further when the information of the time calibration points was added to the phylogeny inference (see below).

### (c) Time calibration of the phylogeny

The time-calibrated phylogeny showed the MRCA of Loricarioidei at 123.8 Ma (figure 2; electronic supplementary material, table S8). The split between the Nematogenyidae and Trichomycteridae families was estimated at 114.0 Ma and the split between the Callichthyidae and the clade containing Loricariidae, Astroblepidae and Scoloplacidae was estimated at 117.2 Ma. The Loricariidae split from the clade including the Astroblepidae and Scoloplacidae at 97.4 Ma, and the Astroblepidae and Scoloplacidae families split at 92.1 Ma. In addition, the MRCA of the Siluriformes order was placed at 146.7 Ma, and the split between the Diplomystidae family and the Siluroidei was at approximately the same time (142.2 Ma).

The time calibration analysis also searches for the best topology and uses the information of the calibration points to make better inferences of the phylogeny. The consensus topology within Loricarioidei after 25% data burn-in is identical to the one obtained with the same dataset with ML and BI methods, and the statistical supports of all inter-familial relationships within Loricarioidei increased to a maximum (PP = 1; table 1 and figure 2).

### (d) Ancestral states reconstruction

The results from the reconstruction of the ancestral states of trunk dermal bony plates and odontodes are summarized in figure 2. Trunk dermal bony plates were not present in the ancestor to all Loricarioidei (probability of absence = 0.82). This trait emerged later on, and is firmly present in the MRCA of the CSAL clade (probability of presence = 0.97) and its descendants. However, dermal bony plates were probably lost in the MRCA of the Scoloplacidae and Astroblepidae (probability of absence = 0.80) and regained in the Scoloplacidae family. In the outgroup, trunk dermal bony plates emerged in the MRCA to the Doradidae, Auchenipteridae and Aspredinidae (probability of presence = 0.72), but were presumably lost in the MRCA of the Doradidae and Auchenipteridae families (probability of absence = 0.74) and regained in the Doradidae family. The other two siluroid families with dermal bony plates, the Amphiliidae and the Sisoridae, gained this trait independently.

Based on model 1 of odontode evolution, in which gaining odontodes is equally probable regardless of the location in which these odontodes emerge, the ancestor to all Loricarioidei already had odontodes on its trunk. The probability of this is 0.83, and includes the probabilities of the ancestor having odontodes on both the head and trunk (0.66), and only on the trunk (0.17). This finding agrees with the results obtained with model 2 of odontode evolution, the model in which the emergence of odontodes on the trunk had a distinct probability from the emergence of odontodes on the head. Under model 2, the probability that the MRCA to all Loricarioidei had odontodes on the trunk is 1, including the probability of having odontodes on both head and trunk (0.44), and the probability of having odontodes only on the trunk (0.56).

## 4. Discussion and conclusion

In this work, we studied the emergence of trunk dermal bony plates and odontodes in the Loricarioidei suborder to test the hypothesis that dermal bony plates are a necessary prerequisite for the activation of odontogenesis in the trunk. To this end, we inferred a robust phylogenetic reconstruction based on a new, 10-gene molecular dataset and on eight fossil calibration points and then inferred the ancestral states of these structures throughout the loricarioid tree.

### (a) Inter-familial relationships within Loricarioidei and ages of major splits

After mitigating the effects of phylogenetic noise by translating coding sequences, eliminating the fastest-evolving nucleotide sites of non-protein-coding genes and adding time calibration points to aid in the tree inference, we obtained a phylogeny that organized the loricarioid families as presented in figure 2. This arrangement has two notable points: the recovery of a basal clade composed of the Trichomycteridae plus Nematogenyidae families, and the recovery of a sister clade to Loricariidae composed of the Scoloplacidae plus Astroblepidae families. The first point is not new in the field of siluriform phylogeny, as it has been proposed several times based on morphological characters [17,19,30,32,50], and once using molecular data, albeit with very low support [51]. Despite this, the sister group relationship between nematogenyids and trichomycterids has been contested [18,30]. This discrepancy may be because phylogenetic artefacts due to signal saturation are masking the true relationship. Indeed, we found the Nematogenyidae at the root of Loricarioidei when analysing only nucleotide data using BI (DS1, electronic supplementary material, figure S1), and fast-evolving nucleotide sites are known to saturate quickly [37]. Support for the sister grouping of Nematogenyidae and Trichomycteridae becomes stronger as noise due to saturation is reduced through the datasets, from DS1 to DS4, and reaches a maximum of PP = 1 when the information of the time calibration points is added (table 1; electronic supplementary material, figure S1). Hence, we show here the first instance in which a consensus is reached, with very high statistical support, between the molecular and morphological phylogenetic hypotheses regarding the relationship between these two families.

The second novelty in our phylogeny is the grouping of the Scoloplacidae and Astroblepidae. This study is the only instance in which this relationship has been found among both molecular and morphological phylogenetic studies. Other studies usually recover the Scoloplacidae as sister to a clade that includes the Loricariidae and the Astroblepidae (e.g. [18,19]), but we did not find significant support for this relationship in any of our datasets (BS on DS1 = 33, DS2 = 7, DS3 = 13 and DS4 = 8; table 1). In addition, support for the new grouping proposed herein shows a tendency to improve as phylogenetic noise is reduced, with a complementary drop in support for the classical grouping of Loricariidae with Astroblepidae, and when the time calibration information is added to the phylogenetic inference, this support becomes absolute (PP = 1; table 1). This study is the first time that the relationships among all loricarioid families have been studied with more than two genes and with time calibration, and thus this unexpected relationship represents the best molecular hypothesis available to date for these families.

Importantly, our ancestral state reconstructions remain valid even when considering previous phylogenetic hypotheses such as an earlier-branching Nematogenyidae instead of one grouped with Trichomycteridae or an Astroblepidae grouped with Loricariidae instead of Scoloplacidae (see electronic supplementary material).

To date, three ages have been proposed for the siluriform MRCA, with Nakatani *et al*. [52] placing it at 180 Ma, Near *et al.* [53] at 106.1 Ma and Chen *et al.* [44] at 97 Ma. Our result of 146.7 Ma falls in the midpoint between these dates (electronic supplementary material, table S8). As for the MRCA to all Loricarioidei, Nakatani *et al*. [52] place it at 162.1 Ma, while Near *et al.* [53] place it at approximately 90 Ma. We place the MRCA to all Loricarioidei at 123.8 Ma, which is during the early stages of the separation of South America and Africa [42,43].

Interestingly, there are large differences in species richness among Loricarioidei families that cannot be explained by family age only, and perhaps may be linked to a selective advantage of having a body protected by an exoskeleton (see electronic supplementary material).

### (b) Ancestral reconstruction of siluriform trunk dermal bony plates and odontodes

Our results show that in Siluriformes, trunk odontodes emerged before trunk dermal bony plates, as the MRCA of all Loricarioidei had trunk odontodes but not such bony plates. We also show that the loricarioid trunk odontodes were gained and never lost in the approximately 120 million years since the emergence of the clade. Trunk dermal bony plates emerged later, and twice within the Loricarioidei: in the MRCA of the CSAL clade, and then in the MRCA of the Scoloplacidae family. Dermal bony plates also emerged independently four times within Siluroidei. In the Loricarioidei, the gain or loss of dermal bony plates did not seem to have had an effect in the presence of trunk odontodes.

These results answer the initial question of this study and provide evidence that the evolutionary emergence of an underlying dermal bony plate was not a prerequisite for activating odontogenesis in the trunk of the ancestral loricarioid.

### (c) Potential links between trunk odontogenesis and underlying bone

When analysing loricarioid specimens, we noticed that, in species without dermal bony plates, trunk odontodes still grow in close association with other bony structures, such as highly ossified fin spines, or fin rays (figure 1*b*,*f*). In some species of the Hypoptomatinae subfamily of the Loricariidae, odontodes form directly on the exposed coracoid bone of the pectoral girdle [54]. Interestingly, the type of ossification of the bone underlying the odontodes does not seem to be of importance, as dermal bony plates and fin rays ossify intramembranously, and the coracoid ossifies endochondrally [55]. In this sense, it seems that any underlying bone, not just dermal bony plates, may be necessary to trigger odontogenesis in otherwise non-odontogenic trunk tissue. Indeed, the osteogenic and odontogenic gene regulatory networks interact and have elements in common [56–60], and some of the cues used during bone formation may have played a role in enabling the deployment of the odontogenic pathway in the trunk of the ancestral loricarioid.

However, this potential morphogenetic interaction alone does not entirely explain why the loricarioid trunk is capable of producing dental structures. In addition to cues for odontode formation, these regions must have an odontogenic potential, which does not seem to be the case in most extant vertebrate lineages, and indeed in other non-loricarioid catfish. Knowing that dental structures have always been associated with the contribution of the NC, we suggest that the loricarioid trunk is populated by NC cells of uncertain origin with a higher potency than those found in the trunk of most current-day vertebrates.

What exactly is needed to unlock odontogenesis in the vertebrate trunk is a question that can be approached from both an evolutionary and a developmental perspective, and having a living lineage as a case study allows the use of a diversity of experimental tools that are not accessible otherwise. Having access to living individuals and their sequence data enables informative experiments of gene expression or sequence data analysis that can clarify how the loricarioid trunk is able to produce dental structures. For example, studying gene expression throughout embryonic development can reveal whether the odontogenic gene pathway that is deployed to form loricarioid odontodes is the same as the one that is deployed to form their oropharyngeal dentition, and can reveal the upstream genetic changes leading to such a change. Locally blocking certain morphogens that are crucial for bone formation could reveal important interactions between the osteogenic regulatory pathways and the activation of odontogenesis in the trunk. In addition, lineage-tracing studies can identify exactly which cells are producing trunk odontodes, and if there are differences in the migration patterns of NC cells between loricarioids and fish that do not form trunk odontodes.

There are many experimental possibilities, and our study provides fundamental information to develop new hypotheses and guide future approaches for deciphering how dental tissues can be formed in the usually forbidden body region: the trunk.

## Supplementary Material

Supplementary Methods and Results/Supplementary Figure

## Supplementary Material

Supplementary Tables

## Data accessibility

The data and scripts used in this paper can be accessed in: https://genev.unige.ch/research/laboratory/Juan-Montoya and from the Dryad Digital Repository: http://dx.doi.org/10.5061/dryad.2p6b5 [61].
