# Supplementary material for: Trunk dental tissue evolved independently from underlying dermal bony plates but is associated with surface bones in living odontode-bearing catfish
Source: Proc Biol Sci. 2017 Oct 18;284(1865):20171831. doi: 10.1098/rspb.2017.1831 (PMC5666107; doi:10.1098/rspb.2017.1831)
Supplement: Supplementary Methods and Results/Supplementary Figure [file rspb20171831supp1.pdf]

Trunk dental tissue evolved independently from underlying dermal bony plates but is associated to surface bones in living odontode-bearing catfish

Carlos J. Rivera-Rivera[1,2] and Juan I. Montoya-Burgos[1]*

[1] Department of Genetics and Evolution, University of Geneva, Switzerland

[2] Institute of Genetics and Genomics in Geneva (iGE3), University of Geneva, Switzerland

** Corresponding author: juan.montoya@unige.ch*

Electronic supplementary information

Supplementary methods

Histological studies: cryosectioning and imaging

We obtained the specimens used for the histological images from our aquaria. The *Ancsitrus dolichopterus* sample was cleared and alizarin-red stained following standard protocols prior to cryosectioning, the *Corydoras sterbai* sample was freshly collected, the *Astroblepus sp.* sample was obtained from a frozen specimen, and the *Tridensimilis brevis* from specimens fixed in 80% ethanol. All specimens were first re-fixed in paraformaldehyde 4% pH 7.4 for two days, and then washed in phosphate-buffered saline (PBS). Then, the samples were left in a solution of 30% sucrose in PBS overnight, and then on optimal cutting temperature compound (OCT, TissueTek) overnight. The cutting blocks were prepared on dry ice by pouring OCT into moulds made from aluminium foil. Once completed, the cutting blocks were left overnight at -80° C. Cryosectioning was done on a Leica CM1850, and 8 µm sections were placed onto SuperFrost/Plus slides (Assistent), and immediately covered in a solution of polyvinyl alcohol (Mowiol, Sigma-Aldrich) and a glass cover slip, and left overnight to dry. Imaging was done using Nomarski interference contrast with a Zeiss Axioplan 2 microscope fitted with a Leica DFC300 FX camera.

PCR Amplification and Sequencing

The total PCR volume was 50 µl, with 1x Roche PCR reaction buffer and reagents in the following final concentrations: 0.025 U/µl of Roche TaqDNA Polymerase, 200 nM of each primer (400 nM if primer was degenerate), 200 nM of dNTPs, 400 nM of $MgCl_2$, and 1-4 µl template genomic DNA (depending on the extraction's initial concentration). The PCR cycle profile consisted of an initial 94° C denaturation step, followed by 40 cycles of: 30s denaturation at 90° C, 30s primer annealing at 51-62° C, and 1m to 1m30s extension at 72° C. After the 40 cycles, a single 5 min final extension step at 72° was done. Due to the high GC-content of the teleost LSU sequence, we added 3 µl of the detergent dimethyl sulphoxide (DMSO to the PCR mix to improve amplification. Adding DMSO helps to avoid intramolecular secondary structures produced by GC pairing, which can reduce PCR efficiency and hinder the

functioning of the DNA polymerase. The PCR product electrophoresis was done on Midori Green-stained 1.7% agarose gels in 1X Tris/Borate/EDTA (TBE) buffer. Fragments that were difficult to amplify were either purified from gels or cloned into *Escherichia coli* bacteria using Life Techonologies One-shot TOPO-TA Cloning Kit For Sequencing, following the manufacturer's instructions.

Sequencing was done by Macrogen Europe (Amsterdam, the Netherlands), Fasteris (Geneva, Switzerland), or at the sequencing facilities of the Department of Genetics and Evolution of the University of Geneva.

Inputs for time-calibrated phylogeny

The input data were the DS4 alignment partitioned by gene, our best topology for these data rooted following [1] as a starting tree, and the time calibrations with normally distributed priors (electronic supplementary material, table S5). We applied (i) the best substitution model for each gene as calculated previously, (ii) the random local clocks model [2] and (iii) the Yule process of speciation [3,4]. We ran the chain for 30M generations, recording every 1000 steps, and discarded the first 25% of the data as burn-in. Bayesian parameter value distributions were visualised using Tracer v.1.6 [5], and the Markov chain tree data were synthesised into a single tree using TreeAnnotator v1.8.3 [6] by plotting the mean node heights in the maximum credibility tree.

Supplementary results and discussions

Testing the effect of alternative phylogenies in the ancestral state reconstruction

Our final phylogeny differs from previously published hypotheses in two aspects: (i) the placing of the Nematogenyidae sister to Trichomycteridae (some molecular phylogenies place Nematogenyidae as the earliest branching loricarioid), and (ii) the placing of Astroblepidae sister to Scoloplacidae (most phylogenies to date place Astroblepidae sister to Loricariidae). We tested if our results of ancestral state reconstructions were robust even when these previously published phylogenetic arrangements were assumed. For this, we repeated the time-calibration phylogenetic analyses two additional times for 5M generations each, and imposed the respective branching patterns within Loricarioidei. We then obtained the time-calibrated phylogenies and performed the ancestral state reconstruction exactly as before.

Our results were robust to these changes. When the Nematogenyidae are forced to be separated from Trichomycteridae and placed at the base of Loricarioidei, the probability of the ancestral loricarioid having trunk odontodes is 0.86 with Model 1 (0.24 in the trunk only and 0.63 on both head and trunk) and 1 with Model 2 (0.59 in the trunk only and 0.41 on both head and trunk), whereas the probability of having dermal bony plates is 0.08. This trait is then present in the node leading to the clade containing the Callichthyidae, Scoloplacidae, Astroblepidae and Loricariidae (CSAL clade) with a probability of 0.97. Something very similar is obtained when the Astroblepidae are grouped with the Loricariidae instead of with the Scoloplacidae: the probability of the ancestral loricarioid having

odontodes in the trunk is 0.84 with Model 1 (0.17 in the trunk only and 0.67 on both head and trunk) and 1 with Model 2 (0.57 in the trunk only and 0.43 on both head and trunk), and the probability of it having dermal bony plates is 0.17. Again, bony plates emerge later, in the ancestor to the CSAL clade (0.96 probability of presence).

Diversification rates

Interestingly, there are large differences in species richness among Loricarioidei families. A simple explanation may be that older families had more time for speciation, however, the ages of the families contrast with their species richness. The Loricariidae family, for example, diverged from the clade comprising the Scoloplacidae plus Astroblepidae 92.1 Mya, and the diversities in these two groups differ starkly. The total amount of species of the Scoloplacidae and Astroblepidae families combined amounts to 86, whereas in the thoroughly armored Loricariidae family this number reaches 931 species [7]. The Trichomycteridae diversified into more than 250 species in 111.4 Mya, whereas the sister family, the Nematogenyidae, has a single extant species.

The six families composing Loricarioidei represent a small fraction (15%) of the 39 recognized catfish families but contain 40% of all species in the order (1,522 loricarioid species out of a total of 3,763 recognized siluriform species [7]). Most of these loricarioid species are contained into three families, the Loricariidae, the Callichthyidae, and the Trichomycteridae, which together make up 38% of all catfish species. In particular, the Loricariidae family, despite emerging from a relatively young lineage in Loricarioidei, contains almost a thousand species, 223 of which have been discovered in the past ten years [7], suggesting that many more may be waiting to be discovered. A Pearson correlation between family age and number of species confirmed that there is no significant correlation between these two variables (n=6, r=0.0912, p=0.864). It is tempting to hypothesize that the remarkable species richness found in Loricariidae and Callichthyidae families is correlated with a key innovation represented by a complete dermal bony armor covered with odontodes, yet this remains to be demonstrated. This hypothesis, however, cannot be tested due to the small number of existing Loricarioidei families with and without body armour.

References

1. Sullivan JP, Lundberg JG, Hardman M. 2006 A phylogenetic analysis of the major groups of catfishes (Teleostei: Siluriformes) using *rag1* and *rag2* nuclear gene sequences. *Mol. Phylogenet. Evol.* **41**, 636–62. (doi:10.1016/j.ympev.2006.05.044)

2. Drummond AJ, Suchard MA. 2010 Bayesian random local clocks, or one rate to rule them all. *BMC Biol.* **8**, 114. (doi:10.1186/1741-7007-8-114)

3. Yule U. 1925 A Mathematical Theory of Evolution, Based on the Conclusions of Dr. J. C. Willis, F.R.S. *Philos. Trans. R. Soc. B* **213**, 21–87.

4. Gernhard T. 2008 The conditioned reconstructed process. *J. Theor. Biol.* **253**, 769–778. (doi:10.1016/j.jtbi.2008.04.005)

5. Rambaut A, Suchard MA, Xie D, Drummond A. 2014 Tracer v1.6.

6. Drummond AJ, Suchard MA, Xie D, Rambaut A. 2012 Bayesian phylogenetics with BEAUti and the BEAST 1.7. *Mol. Biol. Evol.* **29**, 1969–1973. (doi:10.1093/molbev/mss075)

7. Eschmeyer WN, Fong JD. 2016. Catalog of Fishes, url: http://researcharchive.calacademy.org/research/ichthyology/catalog/SpeciesByFamily.asp. Last accessed: 1/19/2017

Trunk dental tissue evolved independently from underlying dermal bony plates but is associated to surface bones in living odontode-bearing catfish

Carlos J. Rivera-Rivera[1,2] and Juan I. Montoya-Burgos[1]*

[1] Department of Genetics and Evolution, University of Geneva, Switzerland

[2] Institute of Genetics and Genomics in Geneva (iGE3), University of Geneva, Switzerland

** Corresponding author:* *juan.montoya@unige.ch*

Electronic supplementary material, Supplementary Figure S1

Individual family-level phylogenies of Loricarioidei, for datasets DS1-DS4, using (*a*) maximum-likelihood and (*b*) Bayesian inference methods. Bootstrap supports lower than 100 and posterior probabilities lower than one are shown. Note changes in topological arrangements and in node supports as the effect of substitution saturation is progressively reduced from DS1 to DS4. Scale bars represent 0.02 substitutions per site.

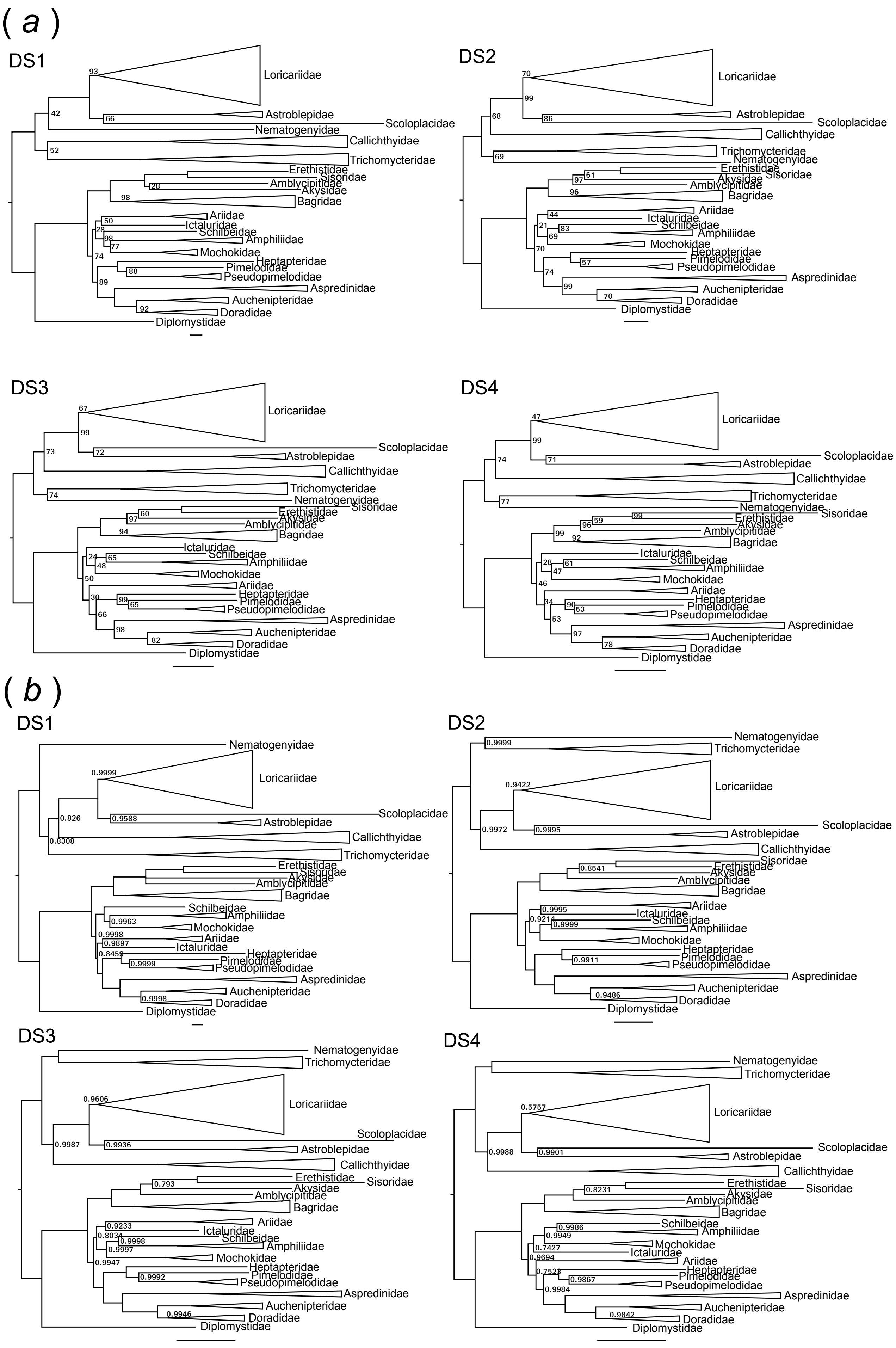
(a)
DS1
93
Loricariidae
42
66
Astroblepidae
Scoloplacidae
Nematogenyidae
52
Callichthyidae
Trichomycteridae
Erethistidae
Sisoridae
28
Amblycipitidae
Akysidae
98
Bagridae
50
Ariidae
Ictaluridae
28
Schilbeidae
98
Amphiliidae
77
Mochokidae
74
Heptapteridae
Pimelodidae
88
Pseudopimelodidae
89
Aspredinidae
Auchenipteridae
92
Doradidae
Diplomystidae
DS2
70
Loricariidae
99
68
86
Astroblepidae
Scoloplacidae
Callichthyidae
Trichomycteridae
69
Nematogenyidae
Erethistidae
61
Sisoridae
97
Akysidae
Amblycipitidae
96
Bagridae
44
Ariidae
Ictaluridae
21
Schilbeidae
83
Amphiliidae
69
Mochokidae
70
Heptapteridae
Pimelodidae
57
Pseudopimelodidae
74
Aspredinidae
99
Auchenipteridae
70
Doradidae
Diplomystidae
DS3
67
Loricariidae
99
73
Scoloplacidae
72
Astroblepidae
Callichthyidae
74
Trichomycteridae
Nematogenyidae
Sisoridae
60
Erethistidae
97
Akysidae
Amblycipitidae
94
Bagridae
Ictaluridae
24
65
Schilbeidae
48
Amphiliidae
Mochokidae
50
Ariidae
30
Heptapteridae
99
Pimelodidae
65
Pseudopimelodidae
66
Aspredinidae
98
Auchenipteridae
82
Doradidae
Diplomystidae
DS4
47
Loricariidae
99
74
Scoloplacidae
71
Astroblepidae
Callichthyidae
77
Trichomycteridae
Nematogenyidae
Sisoridae
99
Erethistidae
59
96
Akysidae
99
Amblycipitidae
92
Bagridae
Ictaluridae
28
Schilbeidae
61
Amphiliidae
47
Mochokidae
46
Ariidae
34
Heptapteridae
90
Pimelodidae
53
Pseudopimelodidae
53
Aspredinidae
97
Auchenipteridae
78
Doradidae
Diplomystidae
(b)
DS1
Nematogenyidae
0.9999
Loricariidae
0.826
Scoloplacidae
0.9588
Astroblepidae
0.8308
Callichthyidae
Trichomycteridae
Erethistidae
Sisoridae
Akysidae
Amblycipitidae
Bagridae
Schilbeidae
Amphiliidae
0.9963
Mochokidae
0.9998
Ariidae
0.9897
Ictaluridae
0.8459
Heptapteridae
Pimelodidae
0.9999
Pseudopimelodidae
Aspredinidae
Auchenipteridae
0.9998
Doradidae
Diplomystidae
DS2
Nematogenyidae
0.9999
Trichomycteridae
0.9422
Loricariidae
0.9972
Scoloplacidae
0.9995
Astroblepidae
Callichthyidae
Sisoridae
0.8541
Erethistidae
Akysidae
Amblycipitidae
Bagridae
Ariidae
0.9995
Ictaluridae
0.9214
Schilbeidae
0.9999
Amphiliidae
Mochokidae
Heptapteridae
0.9911
Pimelodidae
Pseudopimelodidae
Aspredinidae
Auchenipteridae
0.9486
Doradidae
Diplomystidae
DS3
Nematogenyidae
Trichomycteridae
0.9606
Loricariidae
0.9987
Scoloplacidae
0.9936
Astroblepidae
Callichthyidae
Erethistidae
0.793
Sisoridae
Akysidae
Amblycipitidae
Bagridae
0.9233
Ariidae
Ictaluridae
0.8034
0.9998
Schilbeidae
0.9997
Amphiliidae
0.9947
Mochokidae
Heptapteridae
0.9992
Pimelodidae
Pseudopimelodidae
Aspredinidae
Auchenipteridae
0.9946
Doradidae
Diplomystidae
DS4
Nematogenyidae
Trichomycteridae
0.5757
Loricariidae
0.9988
Scoloplacidae
0.9901
Astroblepidae
Callichthyidae
Erethistidae
0.8231
Sisoridae
Akysidae
Amblycipitidae
Bagridae
Schilbeidae
0.9986
0.9949
Amphiliidae
0.7427
Mochokidae
0.9694
Ictaluridae
Ariidae
0.7523
Heptapteridae
0.9867
Pimelodidae
Pseudopimelodidae
0.9984
Aspredinidae
Auchenipteridae
0.9842
Doradidae
Diplomystidae
